# Pectin supplement significantly enhanced the anti-PD-1 efficacy in tumor-bearing mice humanized with gut microbiota from patients with colorectal cancer

**DOI:** 10.7150/thno.54476

**Published:** 2021-02-19

**Authors:** Shi-Long Zhang, Yu-Qin Mao, Zheng-Yan Zhang, Zhan-Ming Li, Chao-Yue Kong, Hui-Ling Chen, Pei-Ran Cai, Bing Han, Tao Ye, Li-Shun Wang

**Affiliations:** 1 Key laboratory of whole-period monitoring and precise intervention of digestive cancer (SMHC), Minhang Hospital, Fudan University, Shanghai, 201199, P.R. China.; 2 Institute of Fudan-Minhang academic health system, Minhang Hospital, Fudan University, Shanghai, 201100, P.R. China.

**Keywords:** colorectal cancer, gut microbiota, pectin, programmed death-1 monoclonal antibody, butyrate

## Abstract

**Background:** Anti-PD-1-based immunotherapy has emerged as a promising therapy for several cancers. However, it only benefits a small subset of colorectal cancer (CRC) patients. Mounting data supports the pivotal role of gut microbiota in shaping immune system. Pectin, a widely consumed soluble fiber, has been reported to ameliorate the imbalance of gut microbiota. Therefore, we aimed to explore the effect and the underlying mechanisms of pectin in improving anti-PD-1 mAb efficacy.

**Methods:** The C57BL/6 mice were treated with a broad-spectrum antibiotic (ATB) cocktail to depleted endogenous gut microbiota and subsequently humanized with feces from healthy controls or newly diagnosed CRC patients. The antitumor efficacies of anti-PD-1 mAb combined with or without pectin were assessed using these mice. Flow cytometry and immunohistochemistry (IHC) were conducted to investigate the tumor immune microenvironment after treatment. The gut microbiota profiles and short-chain fatty acids (SCFAs) levels were determined by 16S ribosomal RNA (16S rRNA) gene sequencing and gas chromatography-mass spectrometry (GC-MS), respectively. The effect of gut microbiota on anti-PD-1 mAb efficacy after pectin supplement was further tested by fecal microbiota transplantation (FMT).

**Results:** The anti-PD-1 mAb efficacy was largely impaired in the mice humanized with feces from newly diagnosed CRC patients compared to those from healthy controls. However, pectin significantly enhanced the anti-PD-1 mAb efficacy in the tumor-bearing mice humanized with CRC patient gut microbiota. Flow cytometry and IHC analysis revealed increased T cell infiltration and activation in the tumor microenvironment of mice treated with anti-PD-1 mAb plus pectin. In vivo depletion of CD8^+^ T cells diminished the anti-tumor effect of anti-PD-1 mAb combined with pectin. 16S rRNA gene sequencing showed that pectin significantly increased gut microbial diversity and beneficially regulated microbial composition. In addition, we identified unique bacterial modules that were significantly enriched in the anti-PD-1 mAb + pectin group, which composed of butyrate-producing bacteria indicative of good response to immunotherapy. Meanwhile, GC-MS showed that pectin altered the level of SCFA butyrate. Furthermore, butyrate, a main product of dietary fiber in gut microbial fermentation, was found to be sufficient to promote T cells infiltration and thus enhance the efficacy of anti-PD-1 mAb. In addition, FMT demonstrated the effects of pectin were dependent on gut microbiota. Importantly, the beneficial effects of pectin were confirmed in the mice humanized with gut microbiota from patient with resistance to anti-PD-1 mAb.

**Conclusion:** Pectin facilitated the anti-PD-1 mAb efficacy in CRC via regulating the T cell infiltration in the tumor microenvironment, which was potentially mediated by the metabolite butyrate.

## Introduction

Immunotherapy has become an emerging way in cancer treatment 1, which utilizes the immune system to produce anti-tumor effects. Monoclonal antibody targeting the programmed cell death protein 1 (anti-PD-1 mAb), as a novel immunotherapy drug, has yielded tremendous promise in several advanced cancers 2. In fact, a majority of patients with colorectal cancer (CRC), ranking as the most prevalent cancers worldwide, would present primary or acquired resistance during anti-PD-1 mAb treatment 3, which seriously hinders its clinical application. Hence, novel strategies are urgently needed to avoid resistance and enhance the anti-PD-1 mAb efficacy.

The human gut microbiota is a diverse ecosystem harboring more than 10^13^ microorganisms, which outnumbers host cells 10-fold 4. Gut microbiota plays a critical role in influencing human health partly via shaping systematic and local immune function. In these processes, several gut microbiota-derived metabolites serve as the key mediators 5. Short-chain fatty acids (SCFAs), for example, the products of dietary fiber fermentation by gut microbiota, participate in stimulating dendritic cell maturation and cytotoxic T lymphocytes activation 6. Recently, several independent studies have showed the important role of gut microbiota in influencing immunotherapy response in patients with melanoma, non-small cell lung cancer, and renal cell carcinoma 7-11, which provides us a new target to influence the anti-PD-1 mAb outcomes. Meanwhile, studies have suggested that altered gut microbiota are closely associated with occurrence and development of CRC 12-14 and CRC patients are characterized by decreased richness and diversity, and significantly disturbed gut microbiota 15. However, it remains uncertain how to modulate these gut microbiota dysbiosis to improve the anti-PD-1 mAb efficacy in clinic.

Pectin is the main soluble fiber extracted from cell walls of plants and it is a natural potential substrate for gut microbiota since it can be fermented by intestine probiotic, resulting in a large number of metabolites 16. These metabolite in turn potentially modulates composition and activity of the gut microbiota in a beneficial way, lower gut pH, for example, to improve the breeding environment for gut flora 16. What is more, pectin and its derivatives can inhibit prostatic cancer, colon cancer by inducing cell cycle arrest, and apoptosis 17, 18. Nowadays, pectin, as a natural food additive, has been widely applied in food and medicine industry. For the scientifically proven health benefits and immunological activities of pectin, we directed our efforts to explore the potential of pectin to enhance anti-PD-1 mAb efficacy in tumor model.

From a clinically translational perspective in future, we established humanized microbiome mice (mice colonized with human gut microbiota), a feasible approach to promote translation of animal study to humans 19. Using this model, we initially investigated whether, and to what extent, the gut microbiota from CRC patients would influence the anti-PD-1 mAb efficacy. Then we tried to explore whether pectin could be used to enhance the efficacy of anti-PD-1 mAb in the context of the human microbiota. The underlying mechanisms involved in alterations of tumor microenvironment, gut microbiota profiles and metabolites were further elucidated. Furthermore, we investigated whether the effect of pectin was mediated by the metabolite butyrate. Our work provided the first basis that pectin could improve the anti-PD-1 mAb efficacy through modulating tumor microenvironment and gut microbiota, which might be expected to open a new avenue for CRC immunotherapy.

## Materials and Methods

### Participants

Five patients newly diagnosed with CRC were included in this study. All CRC patients were confirmed by colonoscopy and histology. Patients would be excluded once they had one of the following conditions 20: 1) use of antibiotic or probiotics at least 3 months before fecal samples collection; 2) had a history of autoimmune and inflammatory diseases and undergoing immunomodulatory therapy; 3) had history of other malignant tumors or undergoing anti-tumor therapy; 4) had history of fecal microbiota transplantation; 5) on a vegetarian diet. Healthy controls were recruited from asymptomatic individuals whose colonoscopy confirmed no significant abnormalities. The exclusion criteria for healthy controls were in accordance with the eligible CRC patients. Additionally, a male patient, aged 70 years, with advanced tumor was also included, whose disease progress after treatment with anti-PD-1 mAb according to the iRECIST criteria 21. The present study was conducted under the ethics committee of Minhang Hospital, Fudan University and informed consents were obtained from all participants.

### Humanized microbiome mice establishment

Six-week-old female C57BL/6 mice, weighing 18.0 ± 2.9 g were purchased from Charles River Laboratories (Hangzhou, China), and maintained under specific pathogen-free (SPF) conditions in a temperature-controlled colony room with 12h light/dark cycle. Mice were fed the rodent chow and water ad libitum. After a one-week adaptation, mice were used for study. Fresh fecal samples from each participant were immediately suspended in an equal volume (w/v) of PBS containing 20% glycerol in PBS, snap-frozen in liquid nitrogen, and stored in -80 °C refrigerator until use. FMT was performed according to the method described previously 22. Briefly, the C57BL/6 mice were gavaged with a broad-spectrum cocktail of oral antibiotics (ATB) containing vancomycin (100 mg/kg); neomycin sulfate (200 mg/kg), metronidazole (200 mg/kg), and ampicillin (200 mg/kg) once a day for three days to remove indigenous gut microbiota. Then, fecal samples were thawed and suspended in an equal volume of PBS, vortexed and centrifuged for the supernatant. One hundred µL of fecal suspension was administered to mice by oral gavage for three days, another 100 µL was applied on the fur of each animal. Within each treatment category, a control group of mice received only PBS. Mice were housed five mice per cage. All experimental protocols were approved by the Ethical Committee of Minhang Hospital, Fudan University.

### Tumor model and treatment regimens

The MC38 colon adenocarcinoma cells (from Type Culture Collection of Chinese Academy of Sciences, Shanghai, China) were cultured in RPMI1640 containing 10% fetal bovine serum (Gibco), 100 U/ml penicillin/streptomycin (BBI Life Sciences Corporation, Shanghai, China) under a humidified incubator, which contains 5% CO^2^ at 37°C. To establish the CRC tumor model, exponentially growing MC38 cells were diluted in 5.0 × 10^5^ cells/ml in PBS and then implanted into the right flank of the C57BL/6 mice. Tumor volume was monitored every three days with caliper, and calculated as length × width^2^ × 0.5. For the anti-PD-1 mAb (Clone: J43; BioXcell, BP0033) group, mice were treated with 200 μg of anti-PD-1 mAb on day 7, 10, and 13 by intraperitoneal injection. For the pectin (D-Galacturonic acid ≥74.0 %, Shanghai Macklin Biochemical Technology Co., Ltd., Shanghai, China, P816453) group, mice were orally gavaged with 10 mg pectin/kg body weight every day since the tumor incubation. For the combination group, mice were treated with anti-PD-1 mAb plus pectin as described above, respectively. For the control group, 200 µg of isotype IgG (Clone:2A3, BioXcell, BE0089) was intraperitoneally administrated to mice at the same time point. The survival of these mice was checked and recorded every day. At the end of the experiment, these tumor-bearing mice were euthanized, and feces, intestines, and tumors were harvested and processed for further analysis.

To determine the role of CD8^+^ T cell, anti-mouse CD8α (BioXcell, BE0117) mAb was applied to deplete CD8^+^ T cells in vivo. In this setting, 200 μg of anti-CD8α mAb was initiated one day before anti-PD-1 mAb administration and continued twice a week for two weeks. To investigate the effect of butyrate on anti-tumor immunity, tumor-bearing mice were provided with 100 mM sodium butyrate (Sigma, V900464) in drinking water for 13 days after tumor implantation.

### Antibiotic treatment and FMT

The tumor-bearing mice were supplemented with pectin at 10 mg/kg in the absence or presence of streptomycin intervention (200 mg/kg). FMT was performed according to a well-established protocol 22. In brief, the donor mice were fed daily with PBS or pectin (10 mg/kg). After 14 days, feces were collected daily from the donor mice. The recipient mice were treated with anti-PD-1 mAb and colonized daily with fresh feces from either PBS or pectin-treated mice. Feces from donor mice were pooled and 100 mg feces were re-suspended in 1 ml of PBS, and homogenized for 1 minute using a vortex to achieve a liquid slurry, and then centrifuged at 8000 g for 3 minutes, and the supernatant was collected and used as transplant material. To minimize microbial population changes, fresh feces were prepared within 10 minutes before use. A total of 100 µl fresh transplant material was gavaged daily to each recipient mice for 10 days.

### Microbial DNA extraction and 16S rRNA gene sequencing

Both human and mice feces were snap frozen with liquid nitrogen and stored at -80 °C until use. The genomic DNA of feces was extracted using the DNA extraction kit (TIANGEN, China) according to the manufacturer's guidelines. The fecal DNA was used as template to amplify the hypervariable regions V3-V4 region of 16S rRNA genes. The PCR amplification was performed in triplicate utilizing the barcoded universal bacterial primers (338 F: 5′-ACTCCTACGGGAGGCAGCAG-3′ and 806 R: 5′-GGACTACHVGGGTWTCTAAT-3′) in a Gene Amp PCR-System 9700 (Applied Biosystems, Foster City, CA, USA). Triplicates were pooled, and the PCR amplicons were sequenced using an Illumina HiSeq platform (Illumina MiSeq, USA).

### Microbiome bioinformatic analysis

Gut microbiota α-diversity was analyzed according to OUT information using QIIME software 23. β-diversity was estimated using Bray-Curtis dissimilarity and the Jaccard similarity index between samples 24. Differential abundance at genus level was identified using R package DESeq2 25. To further analyze differentially-abundant taxa responsible for the classification between two groups, an unsupervised RandomForest classification analysis 26, was performed with the R package randomForest using 1000 trees as well as default settings. Metagenome functional content prediction was performed using PICRUSt (Version 1.1.1) 27.

The R package WGCNA was used to group the genera into modules based on correlation patterns 28. In brief, we used a signed network and chose the minimal β value to meet the scale free topology criteria (optimal β = 6). We adopted a deepSplit value of 3 and a minimum size of 3 for the dynamic tree cut function 29. The co-abundance network was constructed based the relative abundance of taxa from each module, and visualized using Cytoscape 3.5.1 software. Furthermore, the module eigentax was used to detect the correlation between a module and a SCFA or tumor immune parameter. The correlation was determined by the correlation coefficient and *P* value obtained from a univariate regression model between the indicated module and trait. A heatmap plot was used to visualize the results in an intuitive way.

### Fecal SCFAs analysis

The SCFAs were extracted as measured as per the previously described method 30. Briefly, 300 mg feces were homogenized with 1 ml ddH_2_O and centrifuged at 14,000 g for 10 minutes. The supernatant was homogenized with 25% metaphosphoric acid (Aladdin, Shanghai, China) at a volume ratio of 1:1, incubated at room temperature for 4 h, and centrifuged at 12,000 g for 15 minutes. The supernatant was filtered using a 45-μm microporous filtration membrane, and the final concentrations of SCFAs were determined using a gas chromatography-mass spectrometry (GC-MS) system (Agilent 7890/5975C, Santa Clara, CA, United States).

### Immunohistochemistry (IHC) analysis

Freshly isolated tumor issues were fixed in 10% formalin and sliced into 5 μm sections and then used for IHC. The procedure for IHC was performed as described elsewhere 31. The slides were stained with primary antibody as follows: anti-CD4, anti-CD8, and anti-Foxp3.

### Flow cytometry

To characterize the subpopulations of the tumor immune microenvironment, tumors were obtained at the end of study. Tumors were isolated and minced into small pieces and digested in RPMI medium containing collagenase D (1 mg/ml; Roche) and DNase1 (150 UI/ml; Sigma) for 30 minutes at 37°C and then washed and filtered twice using 100 µm cell strainers (Falcon^®^). Fixable Viability Stain 620 (BD Biosciences) was used to discriminate live and dead cells. The cells were then blocked with Fc-block (BD Biosciences) and stained with anti-mouse antibodies against: CD3 (FITC; Biolegend), CD4 (PERCP/Cy5.5 or AF647; Biolegend), CD8a (PERCP/Cy5.5; Biolegend), IFN-γ (AF488; Biolegend), CD25 (APC; Biolegend), Foxp3 (PE; Biosciences), and IL-4 (APC; Biosciences), as per the manufacturer's instructions. Stained samples were analyzed on Cyan ADP 9 colors cytometer (Beckman Coulter) and analyses were performed with FlowJo software version 10.

### Data analysis of patients from The Cancer Genome Atlas (TCGA)

The level-3 RNA-seq data, and clinical characteristics of CRC patients in the TCGA-COAD project were retrieved from cBioPortal (http://www.cbioportal.org/). The CIBERSORT algorithm was applied to estimate the proportions of 22 immune cell subpopulations based on the gene expression data 32. To examine whether CD8^+^ T cells was associated with respond to therapy, we curated the chemotherapy information of all patients from the TCGA-COAD project.

### Statistical analysis

Data was expressed as the mean ± standard error of mean (SEM), and performed with either R (version 3.5.1) or GraphPad Prism 8 (GraphPad, San Diego, CA, USA). Statistical analysis were conducted by unpaired two-tailed Student's t-test. Survival difference was assessed by Kaplan-Meier method and log rank test. *P* < 0.05 was considered to be statistically significant. The significant levels were indicated as follows: *P < 0.05, ** P <0.01, *** P <0.001, **** P <0.0001, ns, no significant difference.

## Results

### Gut microbiota from newly diagnosed CRC patients considerably compromised the anti-PD-1 mAb efficacy in the tumor-bearing mice

**Figure 1:**
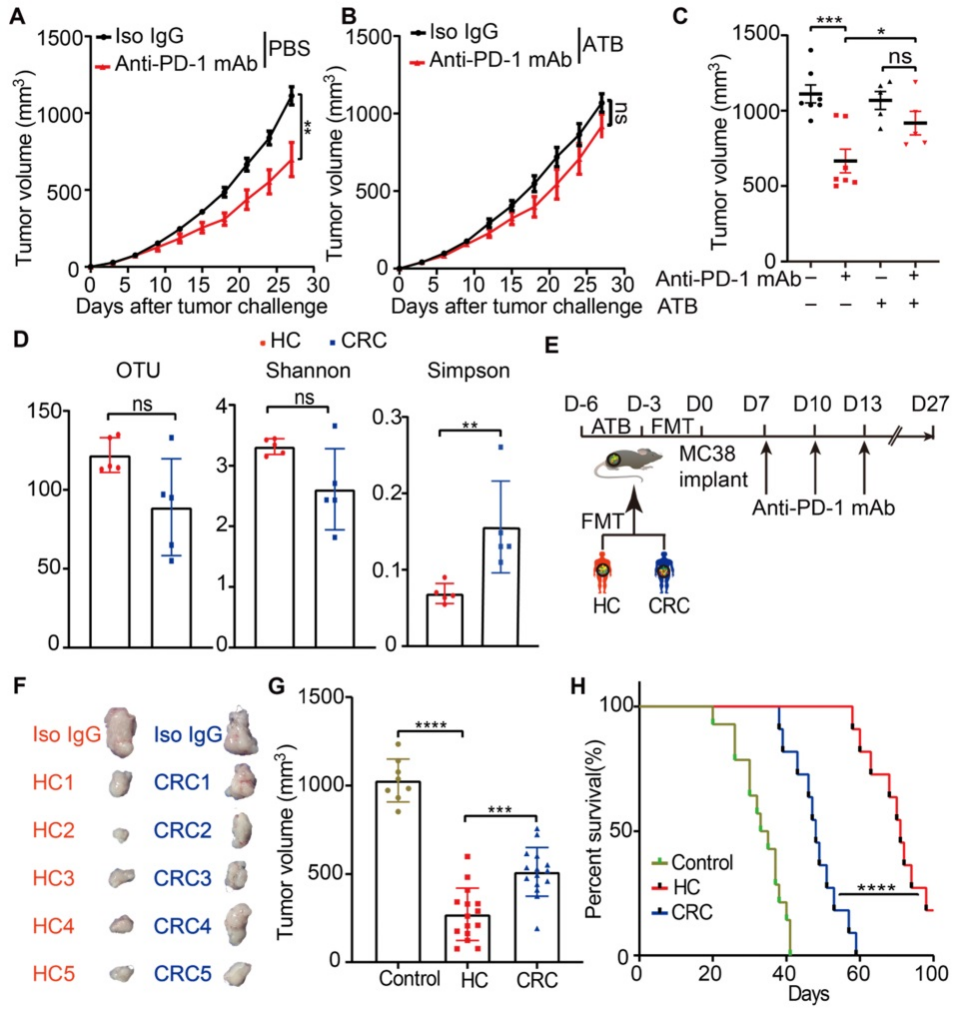
** Gut microbiota from newly diagnosed CRC patients compromised the anti-PD-1 mAb efficacy in the tumor-bearing mice. (A-B)** Tumor growth in the mice treated with isotype IgG or anti-PD-1 mAb in the absence (A) or presence (B) of ATB cocktail (n = 5-8). **(C)** Individual tumor volume were shown as in A-B. **(D)** The α-diversity between the CRC patients and healthy controls were determined using the observed OTUs, Shannon, and Simpson indices (n = 5). **(E)** Experimental design: FMT was conducted after 3 days of ATB cocktail intervention in the SPF mice. Then each mouse was implanted subcutaneously with 5.0×10^5^ MC38 cells and treated with 200 μg of isotype IgG or anti-PD-1 mAb by intraperitoneal injection, every 3 days starting on D7, in total three times. Time in days (indicated as D) relative to tumor injection. **(F)** Representative images of tumor tissues at the end of the experiment. **(G)** The tumor volume of pooled groups of the mice receiving feces from CRC patients or healthy controls (n = 15). **(H)** Kaplan-Meier survival percentage of the mice in each group.

Initially, to test whether the anti-tumor efficacy of anti-PD-1 mAb in CRC was affected by gut microbiota, a syngeneic mouse model bearing with MC38 cells was established. Mice were reared in SPF condition and treated with a broad-spectrum ATB cocktail, a regimen that depleted endogenous gut microbiota 33, or left untreated. Enlarged cecum was found in the mice treated with ATB cocktail, which was a feature of germ-free mice. (**Figure S1A**). ATB cocktail dramatically decreased the α-diversity of gut microbiota (**Figure S1B**) and altered its composition (**Figure S1C**), which indicated basically depletion of gut microbiota. Following a test period of 27 days, ATB cocktail dramatically impaired the tumor control of mice treated with anti-PD-1 mAb (**Figure 1A-C**). This data indicated that the anti-PD-1 mAb efficacy in controlling MC38 tumor growth largely depended on gut microbiota, which is consistent with the observations in MCA-205 sarcoma and RET melanoma mice model 7.

To investigate whether, and to what extent, the disturbed gut microbiota from CRC patients have contributed to the resistance to anti-PD-1 mAb treatment in clinic, the feces from five newly diagnosed CRC patients and five healthy controls were collected in our institute. The detailed characteristics of all participants were listed in the **Table S1**. Then, the gut microbiota profiles of the participants were investigated using 16S rRNA gene sequencing. Compared to healthy controls, the CRC patients were characterized by decreased OTU, Shannon, and increased Simpson indices (**Figure 1D**), although the difference for OTU and Shannon did not reach statistical significance. These data confirmed the disturbance of gut microbiota in the newly diagnosed CRC patients.

Then, we established the humanized microbiome mice with fecal microbiota from these participants using FMT (**Figure 1E**). All recipient mice were well tolerated and survived after FMT. The gut microbiota from CRC patients significantly conveyed resistance to anti-PD-1 mAb in these tumor-bearing mice, compared to the mice receiving microbiota from healthy controls (**Figure 1F-H**). These results might explain the poor response to anti-PD-1 mAb in CRC patients from the gut microbiota perspective.

### Pectin reversed the anti-PD-1 mAb efficacy in the tumor-bearing mice humanized with gut microbiota from CRC patients

**Figure 2:**
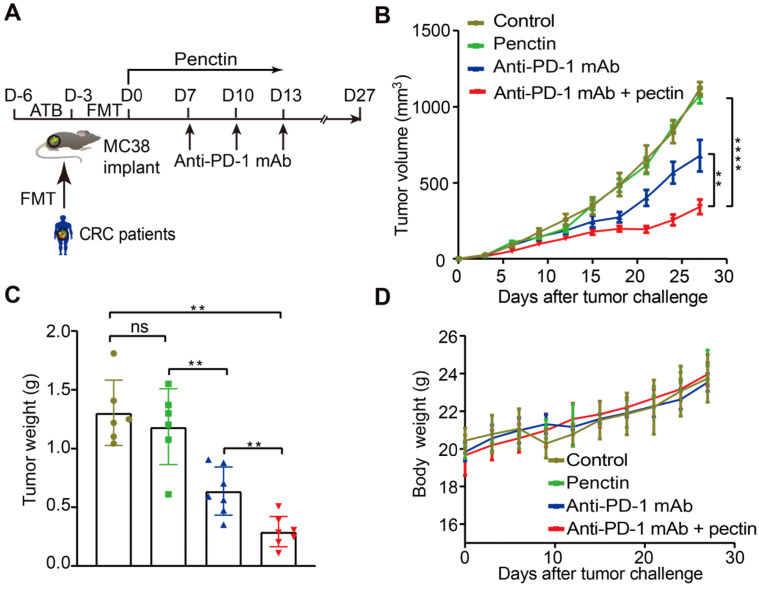
** Pectin enhanced the anti-PD-1 mAb efficacy in the tumor-bearing mice humanized with gut microbiota from CRC patients. (A)** Experimental design: FMT was conducted after 3 days of ATB cocktail intervention in the SPF mice. Then each mouse was implanted subcutaneously with 5×10^5^ MC38 cells and treated with 200 μg of isotype IgG or anti-PD-1 mAb by intraperitoneal injection, every 3 days starting on D7, in total three times. Time in days (indicated as D) relative to tumor injection. For pectin supplement, the mice were gavaged with pectin at a dose of 10 mg/kg from D0 to D13. **(B)** Tumor growth in the mice after treatment with isotype IgG, or anti-PD-1 mAb alone or pectin alone, and anti-PD-1 mAb + pectin (n = 6-9). **(C)** Tumor weight in the mice in each group at the end of experiment. **(D)** Body weight surveillance of each group during the experiment.

Since the gut microbiota from CRC patients dramatically compromised the anti-PD-1 mAb efficacy, we next investigated whether the dietary fiber pectin could reverse the poor efficacy of anti-PD-1 mAb in the CRC patients. Harboring a clinically translational perspective, we established the humanized microbiome mice with feces from these patients as we did above. In order to evaluate the efficacy of anti-PD-1 mAb combined with pectin, we compared tumor growth in these humanized microbiome mice left untreated, or treated with pectin alone, anti-PD-1 mAb alone, and anti-PD-1 mAb combined with pectin (**Figure 2A**). As shown in **Figure 2B**, while pectin alone had no obvious effects on the tumor growth, anti-PD-1 mAb displayed significantly enhanced efficacy when combined with pectin. This result was confirmed by tumor weights from each group (**Figure 2C**). Additionally, the body weight in each group had kept steady increase during the experiment (**Figure 2D**), suggesting that supplement of pectin at 10 mg/kg was relatively safe. Together, pectin could enhance the anti-PD-1 mAb efficacy with a safe profile.

### Combined anti-PD-1 mAb with pectin promoted T cell infiltration and induced CD8^+^ T cell-dependent anti-tumor effect in the tumor-bearing mice

**Figure 3:**
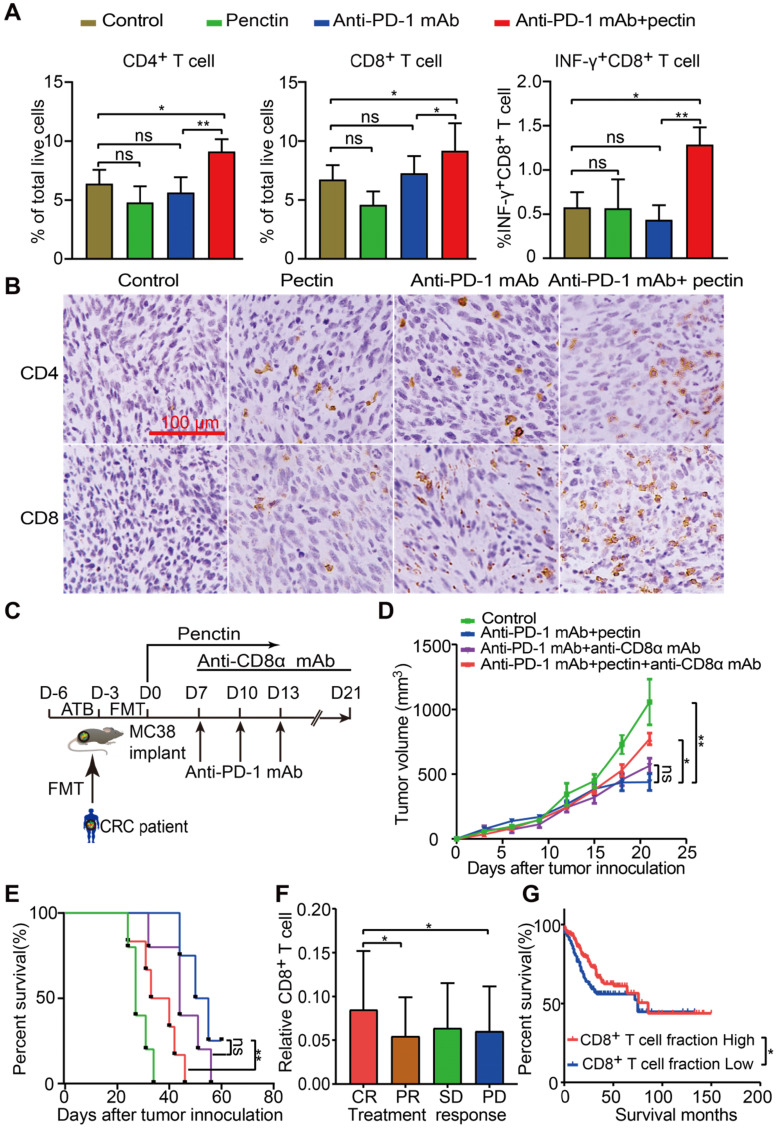
** Pectin supplement combined with anti-PD-1 mAb promoted T cell infiltration in the tumors. (A)** The percentage of CD4^+^ T cell, CD8^+^ T cell and INF-γ^+^CD8^+^ T cell within tumors from the mice in each group at the end of experiment (n = 4-6). **(B)** Representative images of IHC staining for CD4^+^ and CD8^+^ T cell in the tumors from the mice in each group at the end of experiment. **(C)** Experimental design: C57BL/6 mice were implanted subcutaneously with 5.0×10^5^ MC38 cells and treated with anti-PD-1 mAb + pectin. The anti-CD8α mAb was initiated one day before anti-PD-1 mAb treatment and continued twice a week for two weeks. **(D)** Tumor growth in the tumor-bearing mice treated with anti-PD-1 mAb + pectin and depleted for CD8^+^ T cells (n = 5-6). **(E)** Survival time in D, which was defined as the time for tumors to reach a volume of 1500 mm^3^. **(F)** Distribution of CD8^+^ T cell fraction in tumors from the cancer patients with different responses to drug treatment in the TCGA-COAD project. **(G)** Kaplan-Meier survival analysis of progression-free survival according to the fraction of CD8^+^ T cell in tumors from the cancer patients in the TCGA-COAD project. CR, complete response; PR, partial response; SD, stable disease; PD, progressive disease.

Growing data supports that gut microbiota modulates host immune function and tumor responses to therapy 34. To get insights into the molecular and cellular mechanisms behind the beneficial effect of pectin in the anti-PD-1 mAb therapy, we performed flow cytometry to characterize immune cells in the tumor tissues harvested at the end of study. We found that anti-PD-1 mAb + pectin increased the infiltration of T cells, including CD4^+^ T cell and CD8^+^ T cell in the tumors (**Figures 3A**). Importantly, the tumor-infiltrating CD8^+^ T cells also displayed greater effector function, as measured by enhanced production of IFN-γ, a key effector molecule of cytotoxic T cells (**Figure 3A**). T helper type 1 (Th1) cell is a major lineage of CD4^+^ effector T cell that enhances T cell-mediated immunity, and thus can control tumor growth. However, Th1 did not increase in the anti-PD-1 mAb + pectin group (**Figure S2A**). Regulatory T cells (Tregs) are important modulators in tumor progression, and hamper effective antitumor immunity 35. Tregs were decreased in the tumors of anti-PD-1 mAb + pectin group, although they were not significant (**Figure S2B**). Additionally, IHC analysis confirmed the a “hot” tumor microenvironment in the anti-PD-1 mAb + pectin group (**Figure 3B**).

To assess whether the CD8^+^ T cells were required for the favorable phenotype observed in the anti-PD-1 mAb + pectin group, the CD8^+^ T cells were depleted using anti-CD8α mAb. The MC38 tumor-bearing mice were treated as shown in** Figure 3C**, and the depletion efficacy of CD8^+^ T cell was validated (**Figure S3A-B**). As shown in** Figure 3D-E**, anti-CD8α mAb significantly abrogated the antitumor effect of anti-PD-1 mAb + pectin therapy. Hence, CD8^+^ T cell was required to achieve the efficacy observed in the anti-PD-1 mAb + pectin group. Furthermore, to assess whether tumor-infiltrating CD8^+^ T cell could be relevant in humans, we mined gene expression and drug treatment data in the TCGA-COAD project. Using CIBERSORT, we found that CD8^+^ T cell infiltration positively associated with objective treatment response (CR versus PD, *P* = 0.043; CR vs PR, *P* = 0.031; **Figure 3F**), similar to the mouse models. In addition, patients with higher fraction of CD8^+^ T cells enjoyed longer progression free survival than those with low CD8^+^ T cells (**Figure 3G**). Collectively, anti-PD-1 mAb combined with pectin promoted T cell infiltration into the tumor microenvironment and elicited CD8^+^ T cell-dependent anti-tumor effect.

### Pectin ameliorated the gut microbiota imbalance in the tumor-bearing mice humanized with gut microbiota from CRC patients

**Figure 4:**
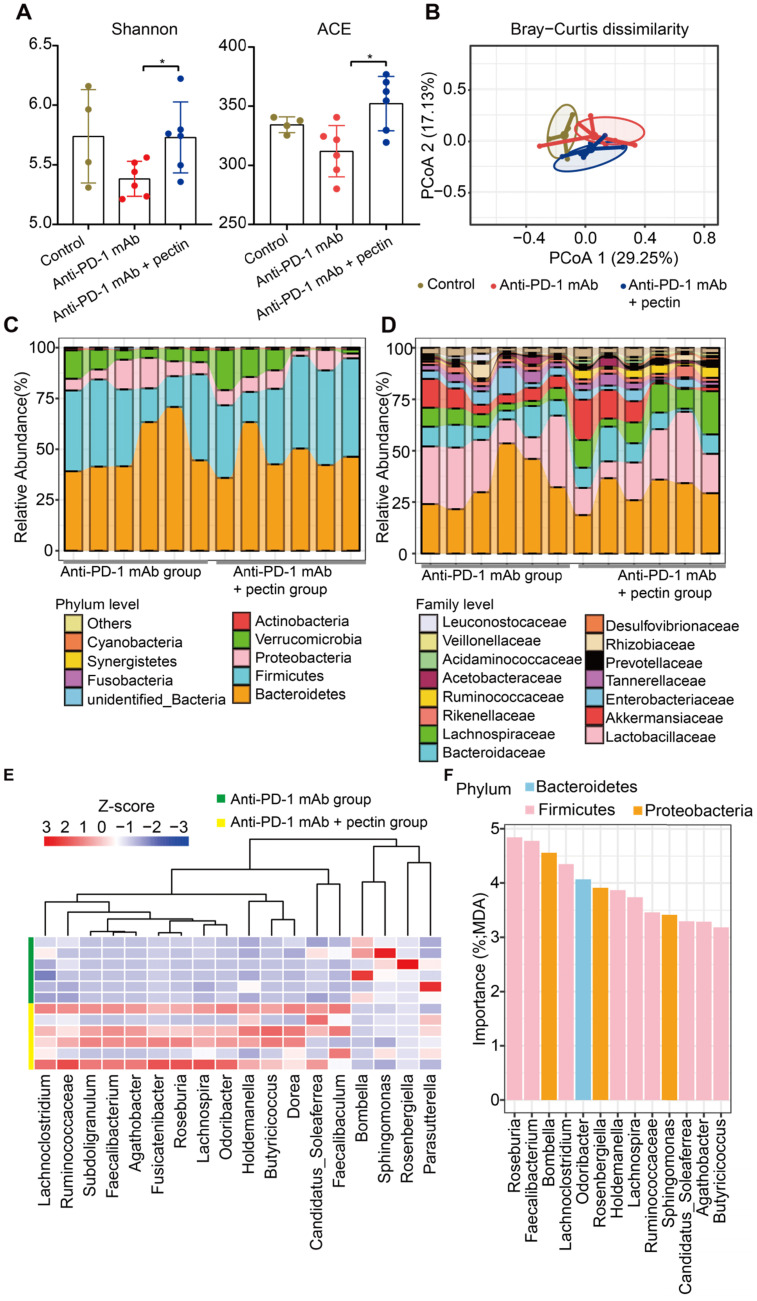
** Pectin supplement modulated the gut microbiota in the tumor-bearing mice humanized with gut microbiota from CRC patients. (A)** Changes in the α-diversity, including Shannon and ACE indices, in anti-PD-1 mAb and anti-PD-1 mAb + pectin groups (n = 4-6). **(B)** PCoA analysis of β-diversity using the Bray-Curtis dissimilarity. **(C-D)** Stacked bar plot of gut microbiota patterns at the phylum (C) and family level (D) in anti-PD-1 mAb and anti-PD-1 mAb + pectin groups. Relative abundance was plotted for each sample. **(E)** Differential abundance of taxa at the genus level between the anti-PD-1 mAb and the anti-PD-1 mAb + pectin groups, as determined by DESeq2. **(F)** Ranking of differentially abundant taxa at the genus level by importance to the gut microbial community, as determined by their MDA using the unsupervised RandomForest analysis. MDA: mean decrease in accuracy, higher MDA indicated greater importance.

To determine whether and how pectin affected the gut microbiota in the humanized mice, we performed 16S rRNA gene sequencing of feces, which were collected from mice on day 27 after tumor challenge. Firstly, microbial α-diversity of each group was analyzed. A significant increase in α-diversity, as indicated by the Shannon and ACE indices, was observed in the anti-PD-1 mAb + pectin group compared to the anti-PD-1 mAb group** (Figure 4A)**. Subsequently, β-diversity analysis using the Bray-Curtis dissimilarity showed the clear segregation of the overall gut community after pectin supplement (**Figure 4B**).

We also detected the specific changes of gut microbiota at different taxonomic levels. At the phylum level, Bacteroidetes was the most predominant phylum in the anti-PD-1 mAb + pectin group, while Firmicutes was the most in the anti-PD-1 mAb group (**Figure 4C**). Additionally, anti-PD-1 mAb + pectin group showed an increase in the Firmicutes-to-Bacteroidetes ratio. At the family level, pectin-treated mice showed partial restoration towards to gut normobiosis (**Figure 4D**). Several well-known microbes used in probiotics, such as Lactobacillaceae, Bifidobacteriaceae, Erysipelotrichaceae, and Ruminococcaceae, which are immunomodulatory SCFA-producing bacteria and associated with healthy gut ecosystems 36-38, significantly increased upon pectin supplement. Differential analysis at the genus level demonstrated increased Ruminococcaceae, Faecalibacterium, and Holdemania in the anti-PD-1 mAb + pectin group (**Figure 4E**), which are reported to be increased in the melanoma patients who responded well to anti-PD-1 mAb 8, 9. Furthermore, among these differential genera, an unsupervised RandomForest analysis showed that Roseburia, Faecalibacterium, Bombella, and Lachnoclostridium ranked as the most important to the gut microbiota, suggesting that pectin exerted more influence on these genera in the gut environment (**Figure 4F**).

### Pectin altered butyrate level in the tumor-bearing mice humanized with gut microbiota from CRC patients

**Figure 5:**
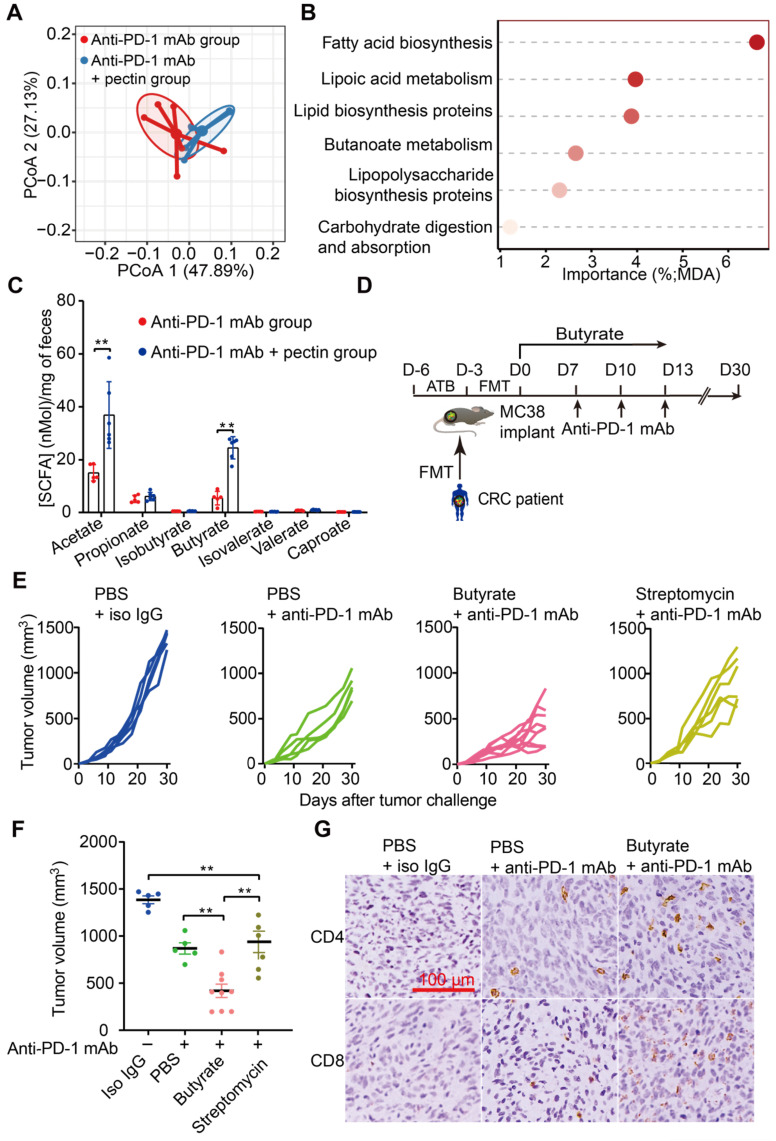
** Butyrate supplement improved the response to anti-PD-1 mAb in the tumor-bearing mice humanized with gut microbiota from CRC patients. (A)** PCoA analysis of KOs between anti-PD-1 mAb and anti-PD-1 mAb + pectin groups, using the Bray-Curtis dissimilarity (n = 6). **(B)** Ranking of KEGG pathways by importance to the gut microbiota function, as determined by their MDA in the unsupervised RandomForest analysis. **(C)** Quantification of SCFAs in fecal samples collected from the mice in the anti-PD-1 mAb, and anti-PD-1 mAb + pectin groups (n = 5-6). **(D)** Experimental design: FMT was conducted after 3 days of ATB cocktail intervention in the SPF mice. After the FMT complement, these mice were administrated with butyrate in drinking water for 13 days. Time in days (indicated as D) relative to tumor implantation. **(E)** Individual tumor growth in the mice after treatment with isotype IgG, or anti-PD-1 mAb alone or anti-PD-1 mAb combined with butyrate, or anti-PD-1 mAb combined with streptomycin (n = 5-9). **(F)** Tumor volume in the mice from E. **(G)** Representative images of IHC staining for CD4^+^ and CD8^+^ T cell in the tumors from E. MDA: mean decrease in accuracy, higher MDA indicated greater importance.

The gut microbiota continually produces dozens of metabolites, and some of these metabolites, particularly SCFAs, influence the host immune response in the gut and beyond 39, 40. Interestingly, gut microbial fermentation of pectin mainly leads to the SCFAs production. Using PICRUSt, we evaluated the metabolic pathways across the anti-PD-1 mAb and anti-PD-1 mAb + pectin groups. PCoA based on KOs revealed a clear segregation of the microbial functional patterns and divergent pathway enrichment between two groups (**Figure 5A**). Furthermore, the RandomForest analysis identified several metabolic pathways, such as fatty acid biosynthesis and butanoate metabolism pathways, that potentially affected by pectin supplement (**Figure 5B**).

We, therefore, hypothesized that these SCFAs might underlie the effects of pectin on tumor immune alterations and enhanced anti-PD-1 mAb efficacy we observed. Thus, we collected mice feces to quantify SCFAs levels using GC-MS. As shown in **Figure 5C**, the levels of acetate and butyrate were significantly increased in the pectin-treated mice, while the other SCFAs were not significantly different among the two groups. Interestingly, supplement with butyrate, prior to anti-PD-1 mAb, was sufficient to rescue the anti-PD-1 mAb efficacy in the non-responders (**Figure 5D-F**). Similar to our above results, IHC analysis showed that this phenotype was accompanied by increased infiltration of CD4^+^ and CD8^+^ cells in the tumor (**Figure 5G**). Furthermore, we depleted the butyrate-producing bacteria using streptomycin, and the butyrate-producing bacteria (**Figure S4A-E**) as well as butyrate (**Figure S4F**) significantly decreased after streptomycin intervention. As expected, the effect of butyrate in facilitating the anti-PD-1 mAb efficacy was abolished (**Figure 5E-F**). In addition, acetate administration did not influence the anti-PD-1 mAb efficacy in controlling tumor growth (**Figure S5**). Collectively, these findings demonstrated that butyrate was the key actuator through which pectin could modulate the efficacy of anti-PD-1 mAb in the tumor-bearing mice humanized with gut microbiota from CRC patients.

### Butyrate-producing bacteria were required for the beneficial effects of pectin

**Figure 6:**
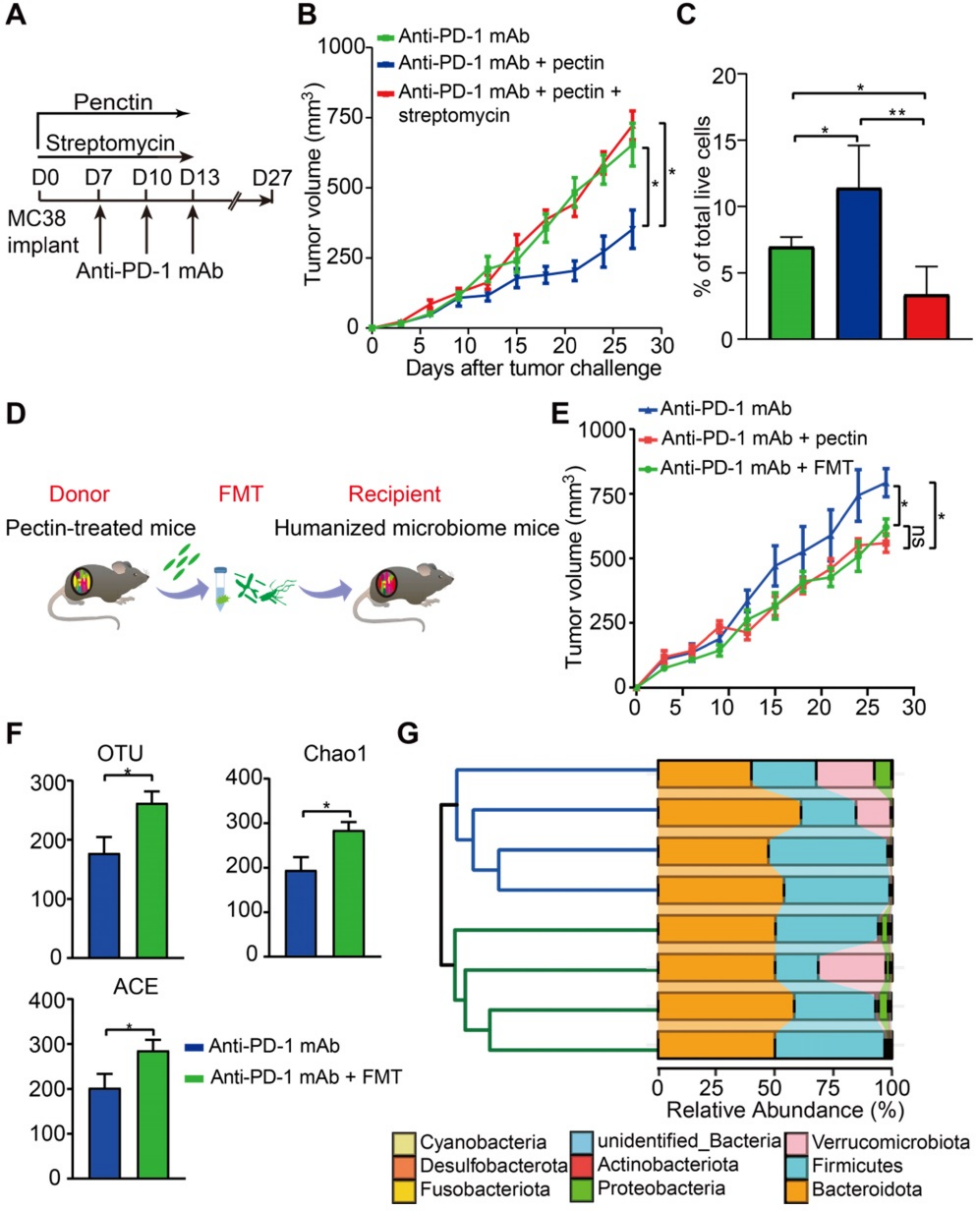
** The beneficial effect of pectin was mediated by gut microbiota. (A)** Scheme for streptomycin intervention. **(B)** Tumor growth in mice treated with streptomycin (n = 5-9). **(C)** The percentage of CD8^+^ T cell in the tumors from A. **(D)** Scheme for FMT. **(E)** Tumor growth in the mice receiving FMT from pectin-treated mice (n = 5). **(F)** Changes in the α-diversity, including observed OTUs, Chao1 and ACE indices, in anti-PD-1 mAb and anti-PD-1 mAb + FMT groups (n = 4). **(G)** Hierarchical clustering tree on the OUT level using the Jaccard dissimilarity (left), and the community map of dominant phyla in each group (right).

To investigate whether the effect of pectin in anti-PD-1 mAb efficacy was dependent on the butyrate-producing bacteria, we treated the MC38 tumor-bearing mice with the streptomycin. Consistent with the above effects on anti-tumor immunity, anti-PD-1 mAb combined with pectin significantly reduced tumor growth. However, when the butyrate-producing bacteria were suppressed by streptomycin, the favorable effect of pectin was greatly diminished (**Figure 6A-B**). Consistently, flow cytometry analysis showed that the increased infiltration of CD8^+^ T cells induced by combination treatment was blocked by streptomycin intervention (**Figure 6C**). These data demonstrated that pectin enhanced the anti-PD-1 mAb efficacy depending on the butyrate-producing bacteria, which in turn influenced T cell infiltration.

### The effect of pectin in enhancing anti-PD-1 mAb was transmissible via FMT

To directly confirm whether the beneficial effect of pectin was mediated by gut microbiota, we performed FMT from pectin-treated mice to the recipient mice for 10 days (**Figure 6D**). As a result, recipient mice displayed significantly improved anti-PD-1 mAb efficacy, as evidenced by the decreased tumor growth (**Figure 6E**).

To evaluate the extent to which FMT modulated the gut microbiota profile, we conducted 16S rRNA gene sequencing of feces, which were collected from mice on day 27 after FMT. FMT from the pectin-treated mice displayed significantly increased α-diversity, including observed OTUs, Chao1, and ACE indices, in the recipient group **(Figure 6F)**. What's more, the hierarchical clustering analysis showed a clear separation of the community structure between two groups, indicating that these communities were distinct (**Figure 6G, left**). The community map reflected the relative abundance and diversity of the phylum in each group, which visually displayed the dominant phyla (**Figure 6G, right**). Collectively, recipient mice recapitulated the similar microbial and anti-tumor efficacy as observed in the donor mice, which indicated that gut microbiota mediated the beneficial effect of pectin in facilitating the anti-PD-1 mAb treatment.

### Pectin altered microbiota networks in the gut

**Figure 7:**
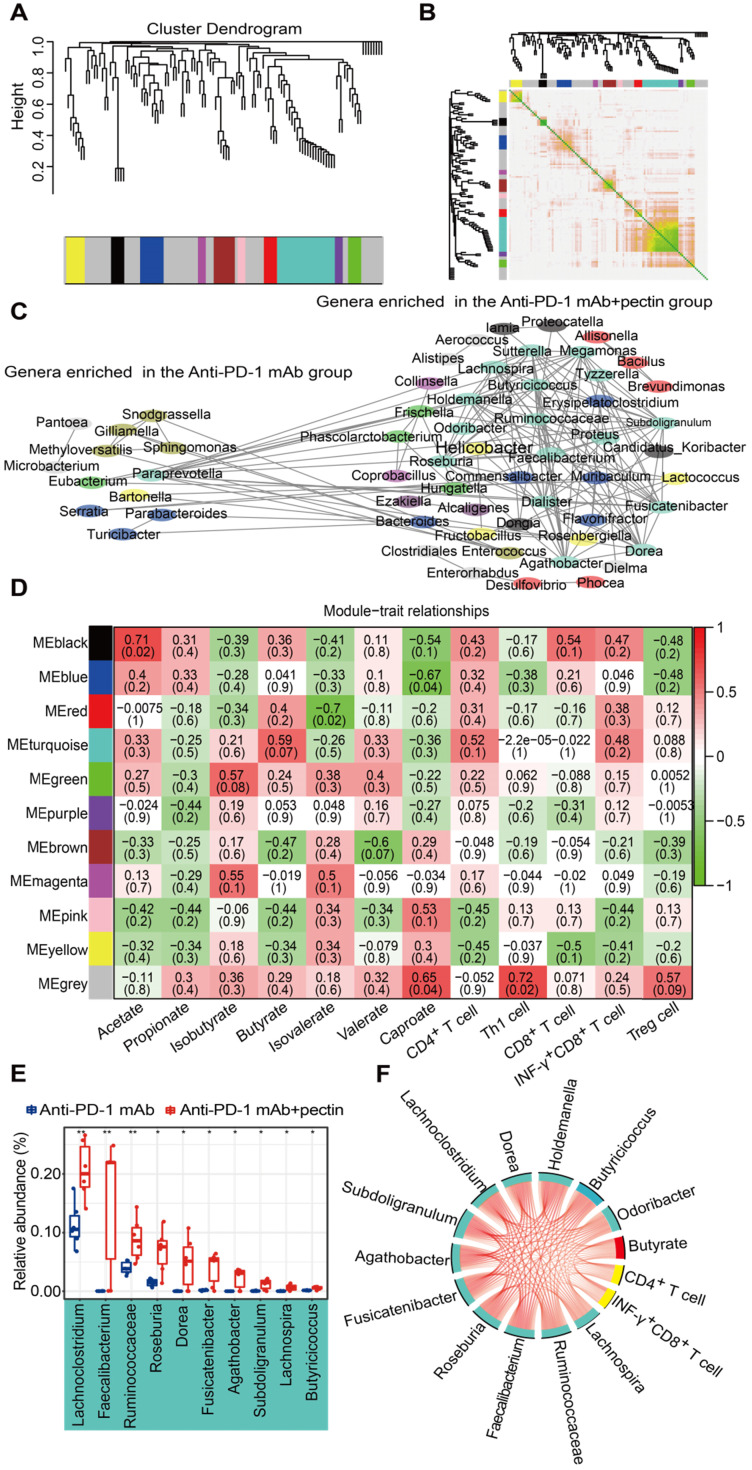
** Gut microbiota co-abundance network at the genus level and module-trait relationships. (A)** Gut microbiota clustering tree at the genus level. **(B)** Interaction analysis of modules at the genus level. The different colors of the horizontal and vertical axes represented different modules. The yellow brightness in the middle indicated the strength of the correlation between the pairs of modules on a linear scale. **(C)** The genus co-abundance network. Genera (nodes) were colored based on WGCNA module colors. Any resulting correlations with *P* ≥ 0.05 and |r| < 0.7 were removed. **(D)** Module-trait relationships were shown. Each cell contained the correlation coefficient between one genus module and a trait in the first line and the corresponding *P* value in the second line. The table was color-coded by correlation according to the color legend (red for positive correlations and green for negative correlations). **(E)** Box-and-whisker plot of the significantly increased genera in the turquoise module. **(F)** Correlation networks of turquoise module, butyrate and tumor immune parameters. Only correlations with a partial spearman's coefficient > 0.7 and *P* < 0.01 were shown.

WGCNA is a new system biological technique, based on microarray or RNA-seq data, that has been more and more used to reveal the relationships between networks, genes, and phenotypic traits 28 and demonstrates high sensitivity to low abundance genes without any information loss 41. Just like genes acting in networks, it is likely that bacterial taxa also function in biological networks in the gut. To better understand the biological implications of taxonomic alterations induced by pectin, we characterized gut microbiota network interactions at the genus levels through WGCNA analysis. As shown in **Figure 7A**, a total of 11 distinct modules of closely associated bacteria were identified within the network when the network soft threshold was 6. The region of color gray represented those bacteria that were not divided into modules. Afterwards, we investigate the interactions among the 11 modules, drew the network heatmap, and demonstrated the independence of the modules (**Figure 7B**). Furthermore, a co-abundance network was constructed based on these modules, and enhanced network density and interactions were observed in the genera that were enriched in the anti-PD-1 mAb + pectin group (**Figure 7C**).

We subsequently assessed the correlations between the gut microbiota modules, SCFA metabolites, as well as tumor immune parameters to further explore the characteristics of the microbiota in the mice treated with anti-PD-1 mAb combined with pectin. The turquoise module, the largest one, was associated with three traits: butyrate, CD4^+^ and INF-γ^+^CD8^+^ T cells (**Figure 7D**); the genera that comprised the turquoise module were mainly involved in the Firmicutes. Moreover, the majority of genera in this module were overlapped with the taxa identified in the differential abundance analysis and significantly upregulated in combined group compared with anti-PD-1 mAb group (**Figure 7E**). Hence, to further outline the mechanisms which mainly modulated by pectin at the systemic levels, we constructed a regulatory network among the taxa in the turquoise module, including SCFA butyrate and CD4^+^ T cell as well as INF-γ^+^CD8^+^ T cell (**Figure 7F**). The network displayed that these factors were strongly correlated with each other, demonstrating the inner association in this circuit.

### Pectin inhibited the tumor growth in the mice humanized with gut microbiome from patient with resistance to anti-PD-1 mAb

**Figure 8:**
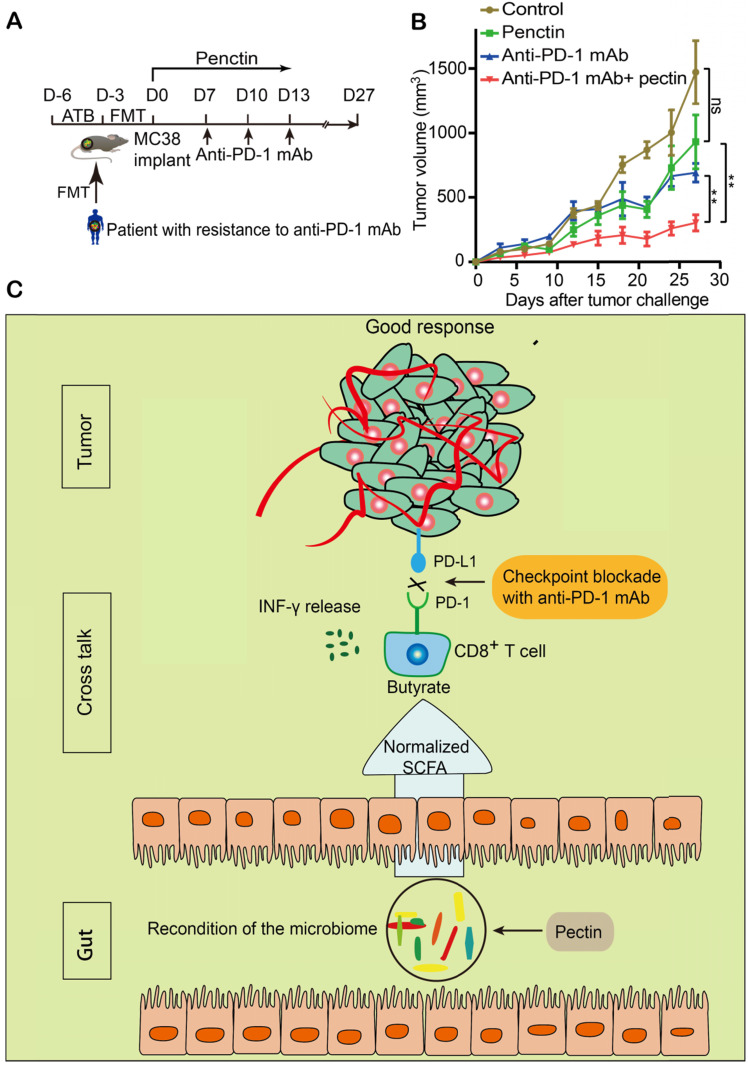
** Pectin supplement enhanced the anti-PD-1 mAb efficacy in tumor-bearing mice humanized with gut microbiota from the patient with resistance to anti-PD-1 mAb. (A)** Experimental design. **(B)** Tumor growth in the mice from each group (n = 5-6). **(C)** Schematic diagram of pectin supplement in the facilitating anti-PD-1 mAb efficacy. Pectin significantly enhanced the anti-PD-1 mAb efficacy in the tumor-bearing mice humanized with gut microbiota from CRC patients, who had dysbiosis of gut microbiome. The improved efficacy was associated with increased T cell infiltration and depended on CD8^+^ T cell, which might be derived from the cross-talk between gut bacteria and host immune system. Furthermore, the SCFA butyrate was the key actuator in this cross-talk.

Based on the successes of pectin (that we applied in the newly diagnosed CRC patients) in enhancing the efficacy of anti-PD-1 mAb, we hypothesized that pectin supplement could be purposed for immunotherapy, since a majority of patients would develop refractory resistance, despite they respond well to anti-PD-1 mAb at first. To address it, we collected the feces from a patient who developed resistance after four cycles of toripalimab, a China-derived marketed anti-PD-1 mAb, and the feces was transferred into cohorts of ATB-pretreated mice (**Figure 8A**). Strikingly, the impaired efficacy of anti-PD-1 mAb were reversed in the mice treated with pectin (**Figure 8B**). Consistent with our analysis above, pectin obviously modified the microbial composition (**Figure S6A**), and increased the α-diversity of humanized microbiome mice (**Figure S6B**). At the same time, the relative abundances of butyrate-producing bacteria were considerably increased in the anti-PD-1+pectin group, confirming the beneficial effect of butyrate in the context of anti-PD-1 mAb (**Figure S6C-H**). To sum up, the phenotype of resistance to anti-PD-1 mAb in the patient could be transferred through gut microbiota. However, pectin could beneficially remodel the gut microbiota dysbiosis, thus reverse the anti-PD-1 mAb resistance.

## Discussion

Colorectal cancer remains one of the most common human digestive malignancies due to its high incidence and poor prognosis 42. Recently, studies of various tumor types have reported that the gut microbiota is a crucial factor associated with efficacy of cancer immunotherapy in both animal models and humans 7-11. Inspired by these findings, we hypothesized that the intervention way targeting gut microbiota for CRC patients might be feasible to facilitate the anti-PD-1 mAb efficacy. Currently, the main approaches to modulate gut microbiota include FMT, direct administration of live commensal species or consortia of bacteria. Gut microbiota is heavily influenced by both genetic and environmental factors, including age, gender, genotype, diet and environmental exposures 43. And different areas usually harbor considerable differences in microbiological environment 15, leading to variations in gut microbiota structure and composition. Since the microbiome dysbiosis of one individual may not necessarily mirror that of another individual, it's not surprising that FMT may not necessarily facilitate tumor control of anti-PD-1 mAb 8. Worse still, FMT exerts influences through complicated ways, making it difficult to predict the FMT outcomes, and the risk of unknown pathogens dispersal after FMT cannot be avoided 44. In addition, numerous concerns require systematic and scientific evaluation, such as doses, administration routes, and long-term efficacy of this therapy 45.

In fact, among all the factors that influence gut microbiota, diet is the most influential and modifiable 46. Emerging evidence supports the interaction between diet-microbiota-cancer 47. Specially, the dietary fibers are all important dietary components to assist the growth of beneficial bacteria, which can protect against cancers and enhance health. Their beneficial effects may derive from the fermentation of dietary fibers into bioactive metabolites 48, and interactions with local and systemic immune responses 49. However, it remains unclear whether, and to what extent, the dietary fibers could be used to modulate the response to anti-PD-1 mAb through gut microbiota in CRC patients.

Pectin, a widely consumed soluble fiber, has been reported to ameliorate the imbalance of gut microbiota, and produce metabolites with local and systemic effects 16, 50. In this study, we initially investigated the role of gut microbiota in the antitumor immune response triggered by anti-PD-1 mAb. Next, by establishing the humanized microbiome mice with feces from newly diagnosed CRC patients, we found that the disturbed gut microbiota from CRC patients were sufficient to result in the poor response to anti-PD-1 mAb. Thus, to improve the response to anti-PD-1 mAb and with a clinically translational perspective in mind, we investigated the potential of pectin supplement to improve the anti-PD-1 mAb efficacy. We found that pectin could beneficially modify the composition and structure of the gut microbiota in the mice recolonized with feces from either patients with newly diagnosed CRC or resistance to anti-PD-1 mAb. More importantly, the anti-PD-1 mAb efficacy was significantly improved by pectin supplement in both settings.

The concept that pectin has similar activity to prebiotics has been confirmed by its ability to ameliorates the gut microecosystem of inflammatory bowel diseases 51, and obesity 52. Consequently, we outlined the profiles of gut microbiota in the mice using 16S rRNA gene sequencing. After pectin supplement, the abundances of the Lactobacillus and Sutterella were significantly upregulated, whereas the Bacteroides was downregulated. The gut probiotics represented by Lactobacillus has been reported to suppress carcinogenesis, enhance immune and alter the lipid metabolism 53. Lachnoclostridium, Ruminococcaceae, Faecalibacterium, and Subdoligranulum are positively related to the pro-inflammatory immune response 54. Faecalibacterium has a high capacity to induce IL-10 release in the dendritic cells and thus modulated T cell-mediated responses 55. More interestingly, we found that almost all of them could ferment dietary fiber into SCFAs, particularly for butyrate 56. Additionally, in melanoma patients, the high abundances of Ruminococcaceae, and Faecalibacterium usually represent better sensitivity to anti-PD-1 mAb 9. These results suggested that pectin could modulate the gut microbiota, and the diversity and the composition of gut microbiota shaped by pectin considerably contributed to the enhanced anti-PD-1 mAb efficacy.

Improving T cell infiltration in the tumor via manipulating gut microbiota is an area of active research, which yet still stays at its infant stage 57. In our study, anti-PD1 mAb treatment induced a modest increase in the percentage of CD4^+^ and CD8^+^ T cells in the tumor beds. Therefore, it might be one of the possible explanations for the poor efficacy of anti-PD-1 monotherapy in our study 58, 59. However, when combined with pectin, anti-PD1 mAb significantly increased the accumulation of CD4^+^ and CD8^+^ T cells in the tumor compared with other groups. Interestingly, pectin, which beneficially modulated gut microbiota, was not able to inhibit tumor growth or influence CD8^+^ T cells infiltration in the absence of anti-PD-1 mAb, indicating its anti-tumor immunity was dependent on anti-PD-1 mAb. In agreement with our study, recent studies have demonstrated that modulating gut microbiota with bacteria or FMT in the absence of immune checkpoint inhibitors fail to affect anti-tumor immune response or tumor growth 7-10. This interesting phenotype could be attributed to involvement of soluble factors since the specific soluble factors, for example, inosine, can work synergistically with immune checkpoint inhibitors to promote CD8^+^ T cells infiltration into tumors 10. In the CD8^+^ T cell depletion experiment, we found the effect of the combination of pectin + anti-PD-1 mAb was dependent on CD8^+^ T cell. Collectively, pectin could reprogram the tumor microenvironment towards to a “hot” status, which in turn rendered tumors sensitive to anti-PD-1 mAb therapy.

To further investigate the mechanisms involved in the beneficial effect of pectin in influencing the anti-PD-1 mAb efficacy, we assessed the levels of SCFAs in the feces from mice and the effect of SCFA on the tumor growth of humanized microbiome mice. We found pectin significantly reversed the decrease of butyrate in mice, indicating the mechanisms underlying pectin might be associated with the increase of butyrate in the gut, which also coincided with enrichment of butyrate metabolic pathways in the PICRUSt analysis. SCFAs are capable of regulating the phenotypes of numerous immune-related cells such as colonic epithelial cells, macrophages, and lymphocytes 60. Specifically, butyrate not only optimizes the function of conventional CD4^+^ T cells 61, but also increases the expression of effect molecules such as IFN-γ, on CD8^+^ T cells 62. In agreement, we found that butyrate was sufficient to induce stronger antitumor immunity in the mice humanized with gut microbiota from CRC patient, who responded poorly to anti-PD-1 mAb. And this favorable effect was associated with more T cells infiltration in the tumor. In addition, the majority of genera that were significantly increased after pectin supplement, such as Lachnospiraceae, were involved in the production of SCFAs, in particular for butyrate. Therefore, the favorable effects of pectin combined with anti-PD-1 mAb were at least due to the immune-modulating property of its metabolite butyrate. Consistent with our results, a latest study has reported that the concentrations of fecal butyrate were higher in the cancer patients responsive to anti-PD-1 mAb than the non-responders, and were positively associated with longer progression-free survival 63. Importantly, by showing that butyrate was sufficient to enhance the efficacy of anti-PD-1 mAb, we provided evidence that butyrate-producing bacteria potentially acted as novel targets in cancer immunotherapy (**Figure 8C**).

In summary, the disturbed gut microbiota from CRC patients significantly compromised the anti-PD-1 mAb efficacy, however, pectin could beneficially regulate the gut microbiota and enhance the anti-PD-1 mAb efficacy in humanized microbiome mice. These effects might be attributed to the CD8^+^ T cells infiltration and SCFA butyrate production. Hence, our study provided a comprehensive perspective to facilitate anti-PD-1 mAb through pectin, which might inspire new developments to aid cancer immunotherapy.

## Supplementary Material

Supplementary figures and tables.

## Abbreviations

CRC: colorectal cancer
Anti-PD-1 mAb: monoclonal antibody targeting the programmed cell death protein 1
ATB: antibiotic
IHC: immunohistochemistry
16S rRNA: 16S ribosomal RNA
GC-MS: gas chromatography-mass spectrometry
SCFA: short-chain fatty acid
FMT: fecal microbiota transplantation
WGCNA: Weighted Gene Co-expression Network Analysis
SPF: specific pathogen-free conditions
FBS: fetal bovine serum
OTUs: operational taxonomic units
PCoA: principal coordinate analysis
PICRUSt: Phylogenetic Investigation of Communities by Reconstruction of Unobserved States
TCGA: The Cancer Genome Atlas
COAD: colon adenocarcinoma
