# Supplementary material for: Pectin supplement significantly enhanced the anti-PD-1 efficacy in tumor-bearing mice humanized with gut microbiota from patients with colorectal cancer
Source: Theranostics. 2021 Feb 19;11(9):4155–70. doi: 10.7150/thno.54476 (PMC7977465; doi:10.7150/thno.54476)
Supplement: Supplementary file 1 — Supplementary figures and tables. [file thnov11p4155s1.pdf]

Pectin supplement significantly enhanced the anti-PD-1 efficacy in tumor-bearing mice humanized with gut microbiota from patients with colorectal cancer

Shi-Long Zhang[1,2a], Yu-Qin Mao[1,2a], Zheng-Yan Zhang[1,2a], Zhan-Ming Li[1,2], Chao-Yue Kong[1,2], Hui-Ling Chen[1,2], Pei-Ran Cai[1,2], Bing Han[1,2], Tao Ye[1,2], Li-Shun Wang[1,2*]

[1]Key laboratory of whole-period monitoring and precise intervention of digestive cancer (SMHC), Minhang Hospital, Fudan University, Shanghai, 201100, P.R. China.

[2]Institute of Fudan-Minhang academic health system, Minhang Hospital, Fudan University, Shanghai, 201100, P.R. China.

Supplementary figures and Tables

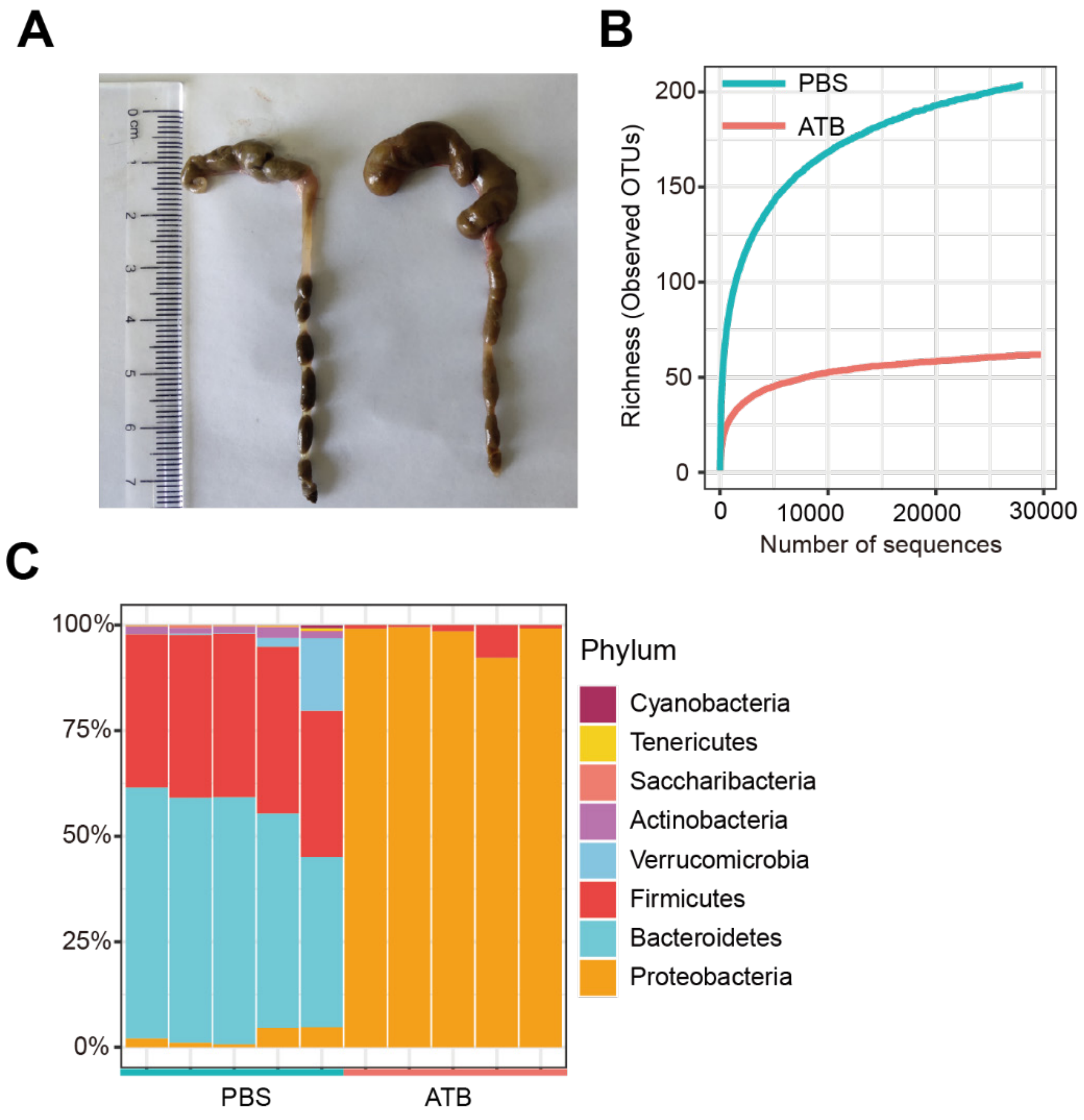

Figure S1. ATB cocktail dramatically decreased the gut microbiota diversity and changed its composition. (**A**) Cecum dissected from PBS-treated or ATB-treated mice. (**B**) Rarefaction curve of PBS-treated or ATB-treated mice were measured. (**C**) Gut microbiota composition at phylum level were compared between PBS-treated and ATB-treated mice (n = 5).

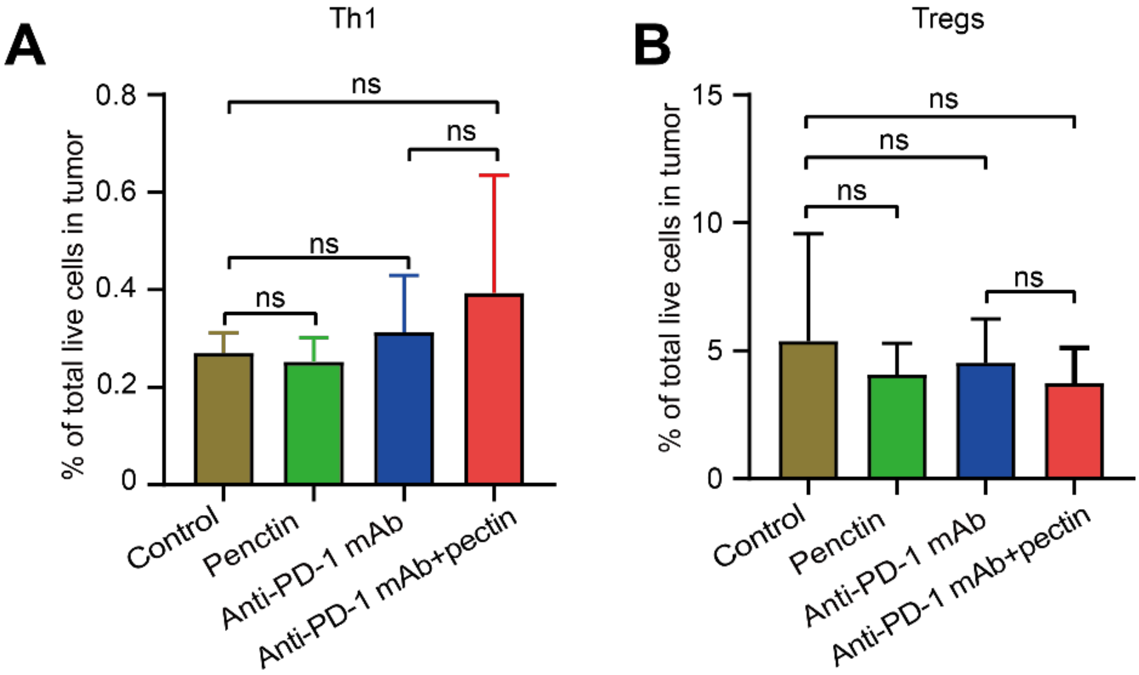

Figure S2. The expression of Th1 cells (**A**) and $CD4^{+}CD25^{+}Foxp3^{+}$ Tregs (**B**) within tumors from Tumor growth from the mice after treatment with isotype IgG, or anti-PD-1 mAb alone or pectin alone, and anti-PD-1 mAb + pectin (n = 3-5).

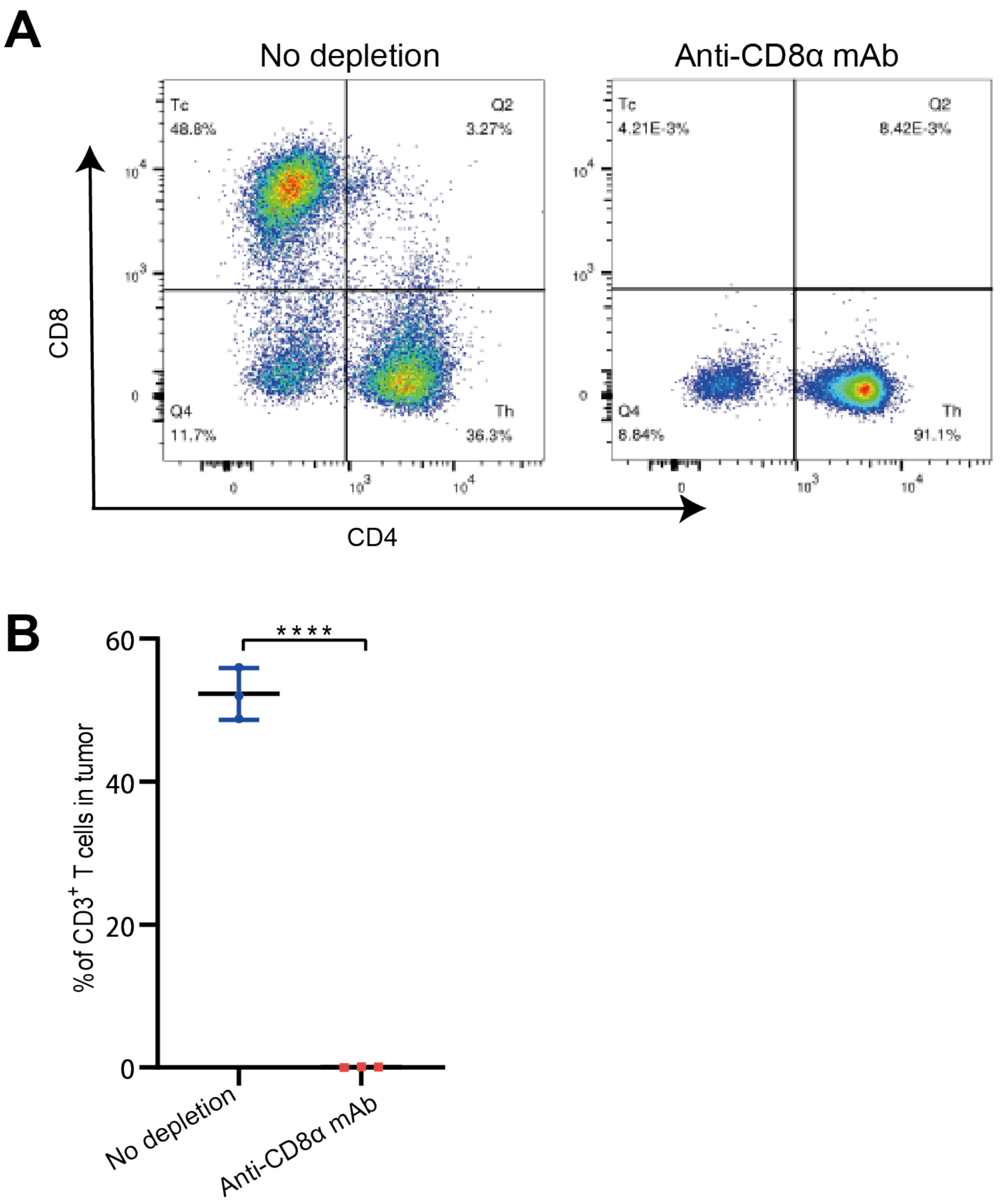

Figure S3. The percentage of $CD8^+$ T cell in the spleen on day 27 after tumor cell inoculation, representative flow data and statistics were showed in (**A**) and (**B**), respectively (n = 3).

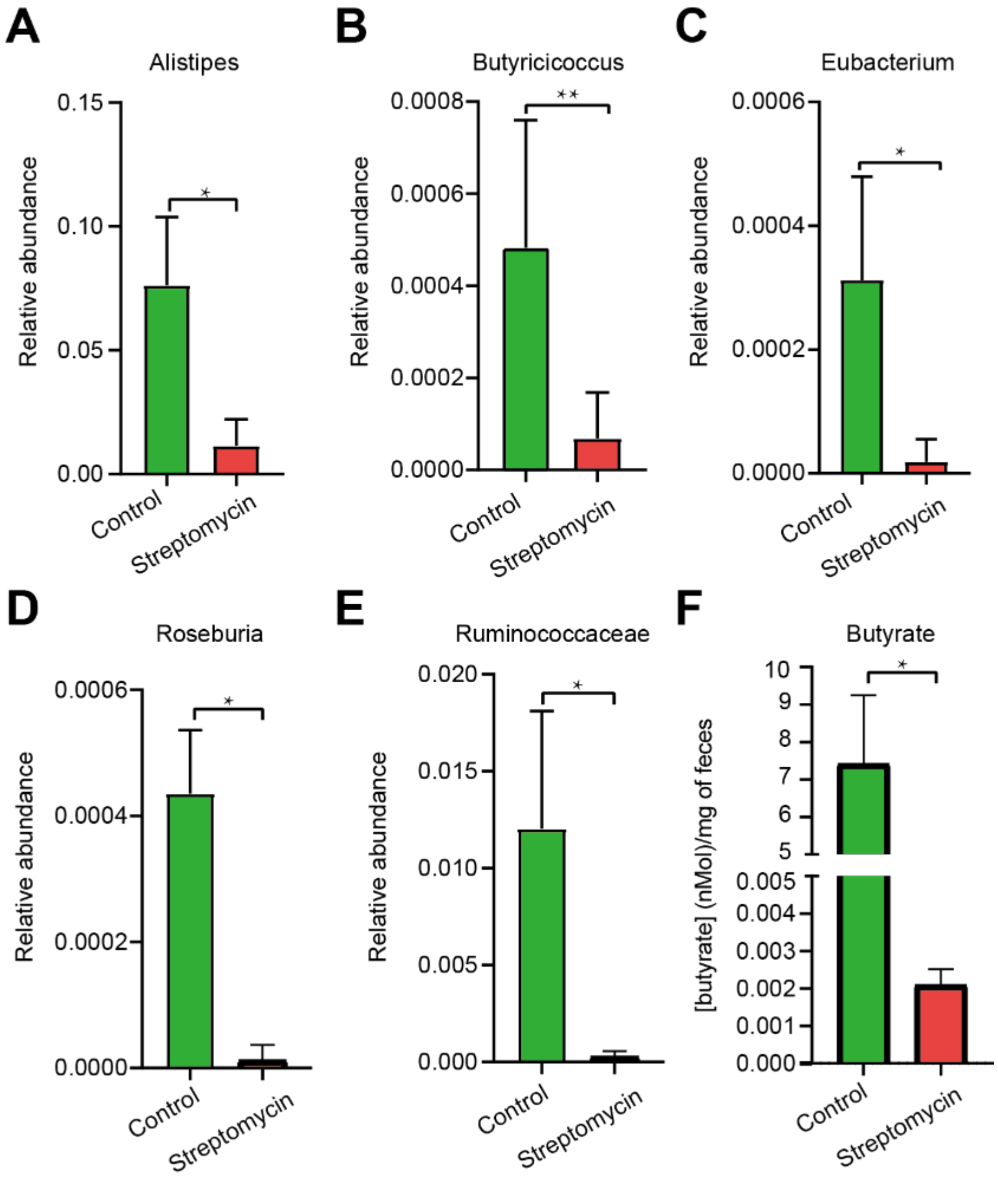

Figure S4. The relative abundance of butyrate-producing bacteria after streptomycin administration (n = 4-5). Each organism depicted is as follows: **(A)** Alistipes; **(B)** Butyricicoccus; **(C)** Eubacterium; **(D)** Roseburia; **(E)** Ruminococcaceae. **(F)** Quantification of butyrate in fecal sample by GC-MS.

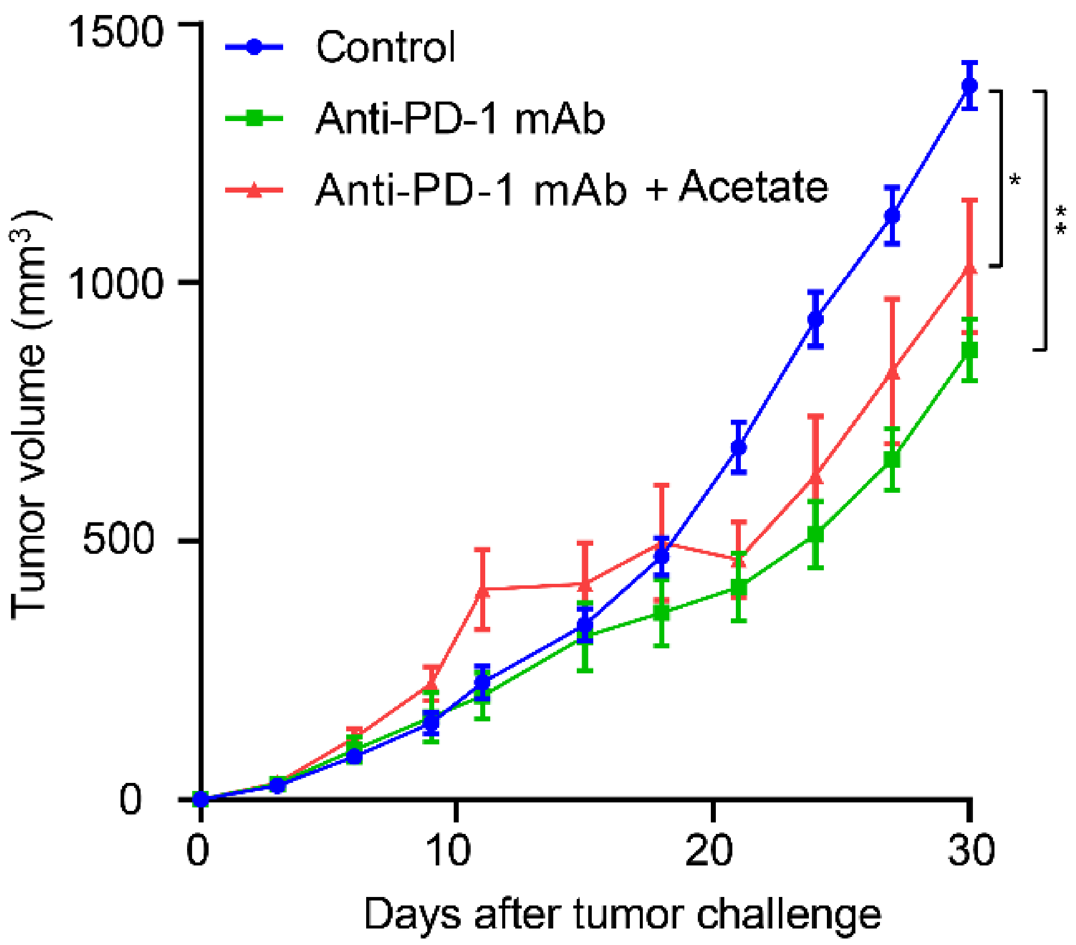

Figure S5. The effect of acetate on the efficacy of anti-PD-1 mAb in tumor-bearing mice (n = 5-6).

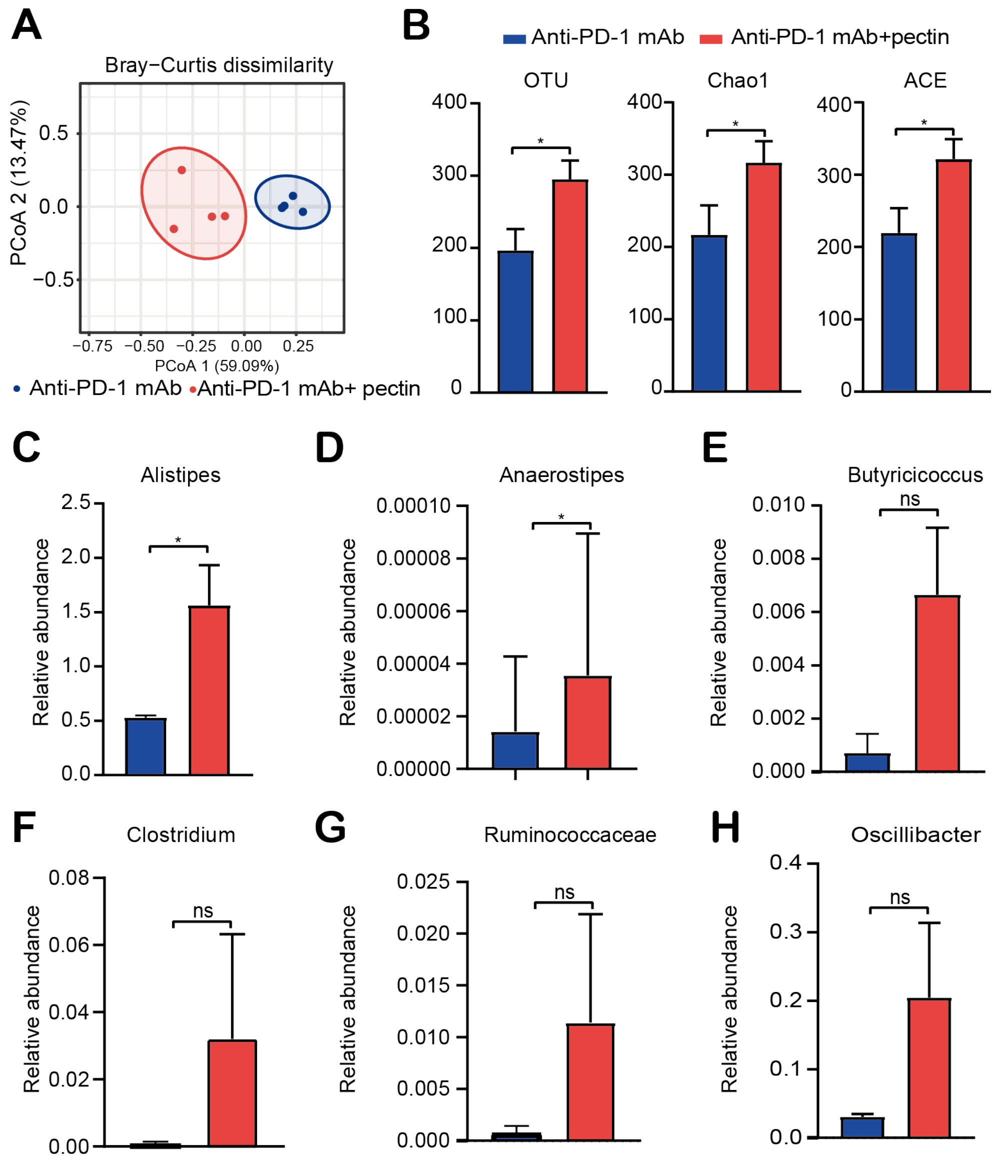

Figure S6. (A) PCoA analysis of β-diversity using the Bray-Curtis dissimilarity (n = 4). **(B)** Changes in the α-diversity indices, including observed OTUs, Chao1, and ACE indices in anti-PD-1 mAb, and anti-PD-1 mAb + pectin groups (n = 4). **(C-H)** The relative abundance of butyrate-producing bacteria in anti-PD-1 mAb, and anti-PD-1 mAb + pectin groups (n = 4). **(C)** Oscillibacter; **(D)** Ruminococcaceae; **(E)** Clostridium; **(F)** Butyricicoccus; **(G)** Anaerostipes; **(H)** Alistipes.

Table S1. Clinical characteristics of the human donors for FMT.

**Group**	**Donor**	**Age**	**Sex**	**BMI (kg/m^2)**	**TNM stage**	**CRC family history**	**Smoking**	**Drinking**	**Dietary preference**
CRC	C1	67	Male	20.3	T4aN0M0	Yes	Yes	Yes	Fatty food
	C2	72	Male	22.1	T4aN0M0	Yes	No	Yes	Normal diet
	C3	67	Male	21.4	T3N1M0	Yes	Yes	Yes	Normal diet
	C4	73	Male	23.7	T4aN0M0	Yes	No	Yes	Sweet food
	C5	62	Male	21.9	T4aN0M0	Yes	Yes	Yes	Fatty food
HC	H1	25	Male	20.7	N/A	No	No	No	Normal diet
	H2	29	Male	20.4	N/A	No	No	No	Normal diet
	H3	26	Male	21.2	N/A	No	No	No	Normal diet
	H4	27	Male	20.9	N/A	No	No	No	Normal diet
	H5	28	Female	20.5	N/A	No	No	No	Normal diet
